# Commendatory

**Published:** 1884-04

**Authors:** 


### COMMENDATORY.

The readers of this journal have the right to know in what estimation it is held by some of those best qualified to judge of its merits. We therefore quote the following from the *Journal of the British Dental Association:*

"Last year saw several minor and two notable additions to the already tolerably long list of dental periodical literature. The INDEPENDENT PRACTITIONER, which had been carried on for three years in the States as a medical journal, with a strong flavor of homeopathy and a little dentistry, finding by experience 'that a journal must have a definite aim, and can satisfactorily serve but one class of readers,' started on a new career, and under energetic editorship * * * has at once taken up a position in the front rank of dental journals. The other important addition is the *Centralblatt fuer Zahnheilkunde,* published in Berlin, and edited by Dr. Golstein, of Geneva."

Although the editor is specially mentioned, those who are acquainted with inside facts know that the success of our enterprise is due more to the energy and ability of its managers, than to the one who stands as the exponent of his associates. They are content to perform unheralded labors for the sake of the profession that they love, while one whom they have pushed forward into the light sometimes receives the credit that is rightfully their due. There is an immense deal of good and hard work done for this journal, aside from its editor's labors.

